# The *Mycobacterium tuberculosis* Phagosome Is a HLA-I Processing Competent Organelle

**DOI:** 10.1371/journal.ppat.1000374

**Published:** 2009-04-10

**Authors:** Jeff E. Grotzke, Melanie J. Harriff, Anne C. Siler, Dawn Nolt, Jacob Delepine, Deborah A. Lewinsohn, David M. Lewinsohn

**Affiliations:** 1 Department of Molecular Microbiology and Immunology, Oregon Health & Science University, Portland, Oregon, United States of America; 2 Division of Pulmonary & Critical Care Medicine, Department of Medicine, Oregon Health & Science University / Portland VA Medical Center, Portland, Oregon, United States of America; 3 Division of Infectious Diseases, Department of Pediatrics, Oregon Health & Science University, Portland, Oregon, United States of America; University of Pittsburgh School of Medicine, United States of America

## Abstract

*Mycobacterium tuberculosis* (Mtb) resides in a long-lived phagosomal compartment that resists maturation. The manner by which Mtb antigens are processed and presented on MHC Class I molecules is poorly understood. Using human dendritic cells and IFN-γ release by CD8^+^ T cell clones, we examined the processing and presentation pathway for two Mtb–derived antigens, each presented by a distinct HLA-I allele (HLA-Ia versus HLA-Ib). Presentation of both antigens is blocked by the retrotranslocation inhibitor exotoxin A. Inhibitor studies demonstrate that, after reaching the cytosol, both antigens require proteasomal degradation and TAP transport, but differ in the requirement for ER–golgi egress and new protein synthesis. Specifically, presentation by HLA-B8 but not HLA-E requires newly synthesized HLA-I and transport through the ER–golgi. Phenotypic analysis of the Mtb phagosome by flow organellometry revealed the presence of Class I and loading accessory molecules, including TAP and PDI. Furthermore, loaded HLA-I:peptide complexes are present within the Mtb phagosome, with a pronounced bias towards HLA-E:peptide complexes. In addition, protein analysis also reveals that HLA-E is enriched within the Mtb phagosome compared to HLA-A2. Together, these data suggest that the phagosome, through acquisition of ER–localized machinery and as a site of HLA-I loading, plays a vital role in the presentation of Mtb–derived antigens, similar to that described for presentation of latex bead-associated antigens. This is, to our knowledge, the first description of this presentation pathway for an intracellular pathogen. Moreover, these data suggest that HLA-E may play a unique role in the presentation of phagosomal antigens.

## Author Summary

Major Histocompatibility Complex Class I (MHC-I) generally serves to present peptides derived from cytosolic proteins to CD8^+^ T lymphocytes, thereby alerting the immune system that the cell is infected. The machinery required for MHC-I antigen processing and presentation is localized to the cytosol and endoplasmic reticulum (ER). After phagocytosis of bacteria, it is unclear how bacterial antigens are presented by MHC-I, as they are segregated from this machinery. In this report, we examine processing and presentation of two proteins derived from the intracellular pathogen, *Mycobacterium tuberculosis* (Mtb). We find that Mtb proteins are able to access the cytosol where they are degraded by the proteasome. Mtb proteins reach the cytosol by retrotranslocation, a process which normally functions to transport misfolded proteins from the ER to the cytosol. Furthermore, the Mtb phagosome contains ER–derived members of the MHC-I peptide loading complex, which aid in loading peptides onto MHC-I molecules. Finally, we detect loaded HLA-I:peptide complexes in the phagosome, demonstrating that loading can occur in the Mtb phagosome. Together, these findings suggest that the Mtb phagosome, through acquisition of ER–derived MHC-I machinery and as a site of MHC-I loading, plays a vital role in presentation of Mtb–derived peptides on MHC-I.

## Introduction

*Mycobacterium tuberculosis* (Mtb) remains a leading cause of morbidity and mortality worldwide, and is the leading cause of death in AIDS patients [1]. Following uptake by phagocytic cells such as macrophages and dendritic cells (DC), Mtb resides in a modified phagosomal compartment with the characteristics of an early endosome [2]. Mtb is able to inhibit phagosomal maturation and lysosomal fusion, and thus survive within the host cell. The cellular immune response is vital for controlling Mtb infection and preventing development of active TB. Macrophage activation through the release of pro-inflammatory cytokines IFN-γ and TNF-α by CD4^+^ and CD8^+^ T lymphocytes is essential for the containment of Mtb [3]. CD8^+^ T cells are uniquely able to recognize intracellular infection, and hence may play a role in immune surveillance through direction of the granule exocytosis pathway to the Mtb-containing cell [4]. In humans, these cellular mechanisms maintain latent TB infection indefinitely in 90–95% of immunocompetent individuals. Thus, the recognition of Mtb–infected cells by T cells is central to prevention of uncontrolled replication. Because the phagosome is a component of the HLA-II processing pathway, the mechanisms by which Mtb-derived antigens are processed and presented on HLA-I molecules remain incompletely understood.

Class I presentation of phagosome associated antigens has been extensively studied using antigen-loaded latex beads. These studies have delineated several distinct presentation pathways [5]. In some cases, phagosomal antigens access the cytosol, allowing processing to occur using the conventional Class I pathway. This pathway is characterized by proteasomal degradation, transport of peptide fragments into the endoplasmic reticulum (ER) by TAP (transporter associated with antigen processing), ER loading onto Class I molecules, and egress of loaded complexes through the ER-golgi [6]. Conversely, antigens can be degraded within a vacuolar pathway, without accessing the cytosol or ER [7]. Cathepsin S is able to generate peptide epitopes in the vacuolar pathway [8] and peptides are subsequently loaded onto potentially recycled Class I molecules [9],[10].

More recently, the phagosome has been implicated in the processing and presentation of phagosomal antigens. Proteins can access the cytosol through the phagosomal acquisition of the retrotranslocation machinery from the ER, which serves to transport antigens into the cytosol [11]. Proteasome generated cytosolic peptides are transported by phagosome-localized TAP into the phagosomal lumen where they are loaded onto Class I (ER-phagosomal pathway) [12],[13]. It has been suggested that ER-derived Class I loading machinery is delivered through ER-mediated phagocytosis and/or subsequent fusion events [14], although there is some controversy regarding this notion [15].

Although the Class I presentation of Mtb antigens is incompletely understood, studies demonstrate the use of multiple pathways [4]. Mtb antigens can be processed via a cytosolic pathway [16], potentially accessing the cytosol through a porous phagosome [17],[18]. Alternately, presentation of lipoproteins or apoptotic bodies derived from mycobacterially-infected DC can be processed through a non-cytosolic pathway [19],[20]. Finally, we have shown that presentation of an antigen presented by the non-classical HLA-I molecule, HLA-E, requires proteasomal degradation but not ER-golgi transport, suggesting that an alternate pathway may be used after proteasomal degradation [21]. These data are consistent with that shown for the ER-phagosomal pathway [13].

In this report, we take advantage of CD8^+^ T cell clones restricted by both classical (HLA-B8 and HLA-B44) and non-classical (HLA-E) Class I molecules to compare the processing and presentation pathway for two Mtb-derived antigens. While both classically and non-classically restricted antigens require cytosolic processing as reflected by dependence on the proteasome and TAP, processing of the HLA-E antigen is distinguished by the possible use of recycled HLA-E. Characterization of the Mtb phagosome reveals the presence of HLA-I, TAP, and protein disulfide isomerase (PDI), and Mtb phagosomes contain HLA-I:peptide complexes. Overall, our data argue that the Mtb phagosome is intimately involved in presentation of Mtb antigens.

## Results

### Mtb antigens access the cytosol but differ in their requirement for ER–golgi transport

To define the molecular requirements for processing and presentation of classically and non-classically restricted Mtb antigens, CD8^+^ T cell clones generated from latent and active tuberculosis patients were utilized. Clone D480 F6 recognizes the 9mer epitope EMKTDAATL from CFP10 (CFP10_3–11_) in the context of HLA-B0801 [22]. D160 1-23 is restricted by the non-classical HLA-I molecule, HLA-E. Although we have not yet defined the antigen or minimal epitope for this clone, it responds to the Mtb cell wall and TX-114 soluble fractions [23]. A CD4^+^ T cell clone (D454 E12) specific for a 15mer containing amino acids 69–83 of CFP10 (CFP10_69–83_ unpublished data) was used as a control.

**Figure 1:**
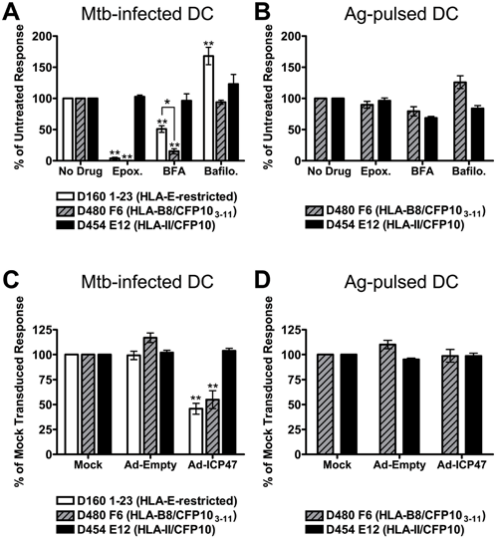
Mtb antigens are processed by the cytosolic pathway. (A,B) Human monocyte-derived DC were treated with epoxomicin, BFA, or bafilomycin for one hour before infection with Mtb H37Rv-eGFP (A) or addition of CFP10 and CFP10_3–11_ (B). After 15–16 hours in the presence of the inhibitor, DC were harvested, fixed, washed extensively, and used as APC in an IFN-γ ELISPOT assay where T cell clones are effectors. DC were added to an excess of T cells so that antigen was the limiting factor (see Materials and Methods). The mean number of spots produced by each clone was: D160 1-23 (228.4±39.3 to Mtb–infected DC, 19.6±6 to uninfected DC), D480 F6 (623.5±73.7 to Mtb–infected DC, 6.7±2.5 to uninfected DC), D454 E12 (453±52.4 to Mtb–infected DC, 13±4.4 to uninfected DC). Data have been normalized to the untreated control, and each bar reflects the mean±SEM of at least three experiments per clone (*p<0.05, **p<0.01 using two-tailed Student's t test compared to untreated controls, except where indicated). (C,D) DC were transduced with either empty vector or adenoviral ICP47 using Lipofectamine 2000. After 6–26 hours, DC were washed and infected with H37Rv-eGFP (C) or pulsed with antigen (D). Following overnight incubation, T cell clones were added and IFN-γ production was assessed by intracellular cytokine staining. The mean percentage of IFN-γ^+^ clones was: D160 1-23 (8.2±1.1 to Mtb–infected DC, 1.1±0.2 to uninfected DC), D480 F6 (46.4±3.9 to Mtb–infected DC, 1.5±0.4 to uninfected DC), D454 E12 (64±6 to Mtb–infected DC, 0.7±0.3 to uninfected DC). Each bar represents the mean±SEM of seven independent experiments.

To evaluate the antigen processing pathway, the effect of inhibitors of proteasomal degradation (epoxomicin), ER-golgi transport [brefeldin A (BFA)], or vacuolar ATPase activity (bafilomycin) on processing of Mtb-derived antigens was assessed. Human monocyte-derived DC were treated with each inhibitor for one hour prior to infection with Mtb. Following overnight incubation in the presence of the inhibitor, DC were fixed and used as antigen processing cells (APC) in an IFN-γ ELISPOT assay using Mtb-specific T cell clones as indicators of antigen presentation. Presentation of CFP10_3–11_ was almost completely inhibited in the presence of epoxomicin and BFA, with no effect of bafilomycin treatment (Figure 1A). This drug inhibition profile is consistent with the cytosolic presentation pathway. To date, analysis of five different Mtb proteins has shown a requirement for cytosolic processing, demonstrating that cytosolic access is not unique to CFP10 (J. Grotzke, manuscript in preparation). HLA-E antigen presentation was also almost completely inhibited with epoxomicin, but only partially inhibited in the presence of BFA (50% inhibition, Figure 1A). These data demonstrate that although the HLA-E antigen requires cytosolic proteasomal degradation, ER-golgi transport is not absolutely required for presentation. Furthermore, bafilomycin treatment actually enhanced HLA-E antigen presentation, suggesting that phagosomal acidification leads to destruction of the epitope.

As a control for drug toxicity and specificity, neither epoxomicin nor BFA interfered with Mtb-derived CFP10 presentation on HLA-II molecules. Also, there were minimal effects of inhibitor treatment on presentation of soluble CFP10 to the CD4^+^ clone or CFP10_3–11_ peptide to the CD8^+^ clone (Figure 1B). Overall, these data indicate that while both classically and non-classically restricted Mtb antigens are processed in the cytosol, these antigens utilize distinct presentation pathways.

### Presentation of Mtb antigens requires TAP transport

The requirement of proteasomal processing argues for cytosolic access of CFP10 and the HLA-E antigen. To determine if TAP transport is required for presentation of these epitopes, we used an adenoviral vector expressing the TAP inhibitor ICP47 [24],[25] and assessed Mtb antigen presentation by IFN-γ intracellular cytokine staining. Infection of DC with adenovirus-ICP47 led to a 25–40% reduction of cell surface HLA-I compared to mock or empty vector transduced cells based on geometric mean fluorescent intensity, while cell surface HLA-II expression was not significantly affected (data not shown). When ICP47 expressing DC were subsequently infected with Mtb, ICP47 inhibited presentation of the HLA-E antigen and CFP10_3–11_ to the HLA-E and HLA-B8 restricted clones, respectively (approximately 50%, Figure 1C). In contrast, there was no ICP47-mediated inhibition of presentation of either soluble or Mtb-derived CFP10 by HLA-II, nor was there any defect in ICP47-expressing cells to present the CFP10_3–11_ peptide (Figure 1D). These data demonstrate that TAP is required for the presentation of these two Mtb proteins.

### Mtb antigens require export out of the phagosome but do not require newly synthesized HLA-E

**Figure 2:**
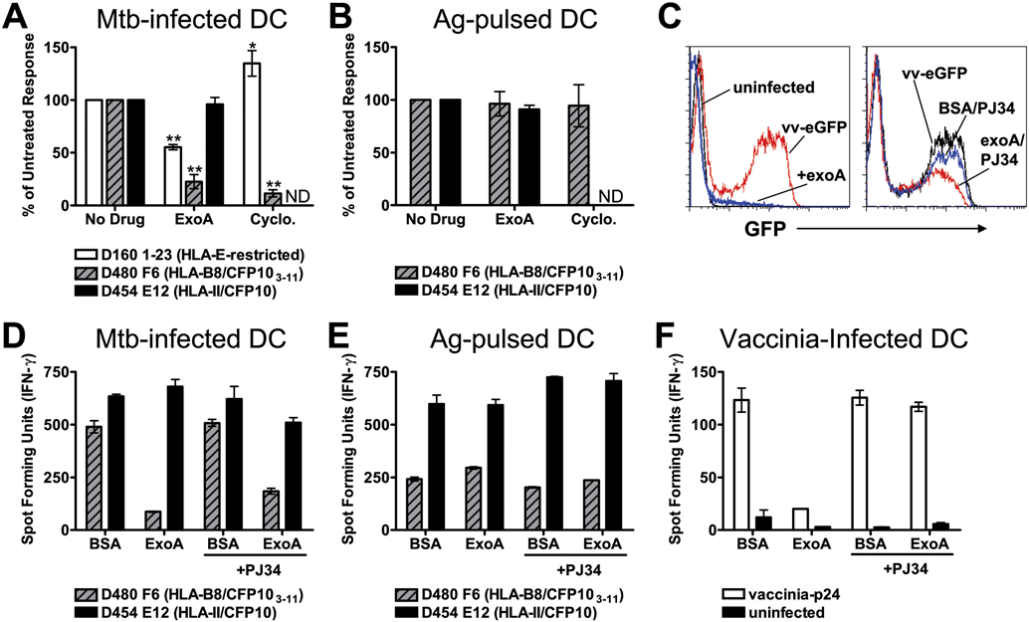
Mtb proteins require retrotranslocation for presentation. (A,B) DC were treated with exoA or cycloheximide for one hour prior to infection with H37Rv-eGFP (A) or addition of CFP10 and CFP10_3–11_ (B). DC were harvested, fixed, and assessed for their ability to stimulate T cell clones by IFN-γ ELISPOT as described. Each bar reflects the mean±SEM of at least three experiments. ND, not done. (C) DC were treated with exoA, exoA/PJ34, or BSA/PJ34 for one hour prior to infection with vaccinia virus expressing eGFP. After 16–18 hours, DC were harvested and GFP expression analyzed by flow cytometry. Data are representative of three experiments. (D,E) DC treated with exoA, BSA, exoA/PJ34, or BSA/PJ34 for one hour were subsequently infected with H37Rv-eGFP (D) or pulsed with antigen (E) overnight, harvested, fixed, and assessed for their ability to stimulate T cell clones by IFN-γ ELISPOT. Data are representative of two experiments. (F) DC were treated with exoA, exoA/PJ34, or BSA/PJ34 for one hour prior to infection with vaccinia virus expressing HIV p24. After 16–18 hours, DC were harvested, fixed and used to stimulate the HIV p24_306–316_-specific CD8^+^ clone 16A7 in an IFN-y ELISPOT assay. Data are representative of two experiments.

The ER contains retrotranslocation machinery that functions to dislocate misfolded proteins into the cytosol where they can be degraded [26]. Recent data show that latex bead phagosomes acquire this machinery and that retrotranslocation is required for cross-presentation of soluble OVA [11]. To determine if Mtb antigens require transport out of the phagosome for cytosolic access, the *Pseudomonas aeruginosa* protein Exotoxin A (exoA) was utilized. ExoA inhibits protein synthesis by ADP-ribosylation of elongation factor-2 [27]. Through interactions with members of the retrotranslocation machinery, exoA is also able to inhibit retrotranslocation [11],[28]. Mtb–infected DC treated with exoA were inhibited by 80% in their ability to process and present CFP10_3–11_, while presentation of the HLA-E antigen was inhibited by 45% (Figure 2A). Because exoA has multiple functions, we sought to determine whether the effect of exoA was due to a block in retrotranslocation or inhibition of protein synthesis. Treatment of DC with exoA led to a dramatic inhibition of vaccinia virus-eGFP expression (Figure 2C), showing that exoA is a potent inhibitor of protein synthesis in primary human DC. Consistent with its effect as an inhibitor of protein synthesis, exoA blocked the presentation of vaccinia-expressed HIV p24 to a p24 specific CD8^+^ T cell clone (Figure 2F). These studies demonstrate that exoA can inhibit general antigen presentation by inhibiting protein synthesis. However, it was not clear whether this mechanism was responsible for the inhibition of Mtb presentation.

The protein synthesis inhibitor cycloheximide was used to examine the effect of protein synthesis on presentation of Mtb antigens. Treatment of DC with cycloheximide inhibited presentation of Mtb-derived CFP10_3–11_ by 90%, while enhancing presentation of the HLA-E antigen (Figure 2A). These data argue two important points. First, exoA inhibition of HLA-E antigen presentation is not due to inhibition of protein synthesis and is likely an effect of retrotranslocation block. Second, newly synthesized HLA-E is not required for antigen loading.

To determine whether retrotranslocation was involved in CFP10 processing, the exoA inhibitor PJ34 was utilized. PJ34 binds to the catalytic domain of exoA with high affinity, thereby inhibiting ADP-ribosylation and abrogating the effect of exoA on protein synthesis [29]. Co-incubation of PJ34 and exoA restored vaccinia virus-eGFP expression to 70% of levels expressed in mock (BSA/PJ34) co-treated DC based on the geometric mean fluorescent intensity, and only 8% fewer cells expressed eGFP in exoA/PJ34 co-treated cells (Figure 2C). Furthermore, presentation of endogenously expressed antigen was indistinguishable from that of BSA or BSA/PJ34 treated controls (Figure 2F). Together, these data confirm that PJ34 blocks the protein synthesis inhibition activity of exoA, and demonstrate that PJ34 itself does not inhibit protein synthesis or endogenous antigen presentation. While not as effective as exoA alone, exoA/PJ34 inhibited Mtb-derived CFP10_3–11_ presentation by over 60%, and had little effect on presentation of Mtb-derived CFP10 by HLA-II (Figure 2D). Presentation of CFP10_3–11_ peptide or soluble CFP10 was not blocked by exoA or exoA/PJ34 (Figure 2B and 2E), further controlling for toxicity of these inhibitors. In total, these data argue that presentation of both Mtb antigens are facilitated by retrotranslocation from the phagosome for cytosolic access.

### The Mtb phagosome contains HLA-I loading accessory molecules

**Figure 3:**
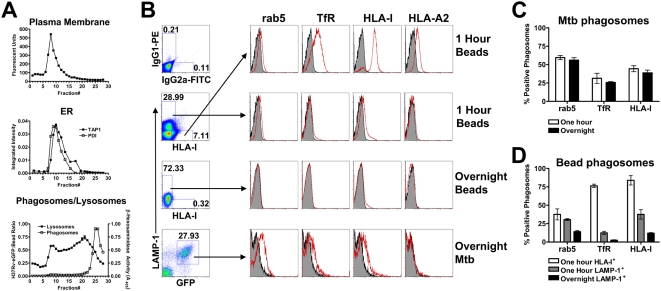
The Mtb phagosome retains characteristics of an early endosome. (A) Representative figure showing organelle distribution after percoll separation of homogenate from Mtb–infected DC. The plasma membrane was labeled with a PE-conjugated antibody to HLA-II prior to homogenization and fluorescence was detected by fluorometry. For detection of ER, fractions were assessed for the presence of TAP1 and PDI by western blot. An enzymatic assay for β-hexosaminidase was used for detection of lysosomes. Finally, fractions were examined for the presence of H37Rv-eGFP by flow cytometry and quantified using a reference latex bead population. For flow cytometric analysis of Mtb phagosomes, the final 2 ml (fractions 23–28) of the gradient were pelleted, fixed, permeabilized, and stained with antibodies of interest. (B) Magnetic bead and Mtb phagosomes were gated based on FSC/SSC (not shown) and then on LAMP-1/HLA-I (beads) or LAMP-1/GFP (Mtb). Arrows indicate the gated population. Analysis of phagosome maturation on one hour LAMP-1^lo/−^/HLA-I^+^ magnetic bead phagosomes (top panel), one hour LAMP-1^+^/HLA-I^lo/−^ magnetic bead phagosomes (second panel), overnight LAMP-1^+^ magnetic bead phagosomes (third panel), and overnight Mtb phagosomes (bottom panel). Plots include isotype staining (shaded histograms) as well as staining with the indicated antibody (red lines). The amplified HLA-I signal on the HLA-I-FITC gated events is due to the use of primary and secondary antibody combination, with which we routinely see up to a log shift in signal over conjugated primary. (C,D) Quantitative analysis of phagosomes over time. The percent positive number represents Overton cumulative histogram subtraction of the isotype control from the indicated stain. Each bar represents the mean±SEM of three experiments per timepoint.

Since our data demonstrate that HLA-E antigen presentation requires TAP transport, yet is insensitive to cycloheximide and only partially blocked by BFA, we hypothesized that peptide loading may occur within the Mtb phagosome. To determine if the Mtb phagosome contains proteins associated with HLA-I peptide loading, flow organellometry was employed. Mtb phagosomes were separated from plasma and ER membranes using a 27% percoll gradient as modified from the protocol of Ramachandra et al. (see Materials and Methods [30]). Figure 3A demonstrates the localization of plasma membrane, ER membranes, lysosomes, and phagosomes in the gradient. While there is some overlap between the phagosomal and lysosomal fractions, phagosomes are clearly separated from the ER and plasma membrane fractions. Phagosome-containing fractions (fractions 23–28) were pooled and used for flow organellometry.

To analyze phagosomal maturation, Mtb phagosomes were compared to phagosomes containing polystyrene-coated magnetic beads at early (20 minute pulse, 40 minute chase, 60 minute total) and late timepoints (20 minute pulse, 18 hour chase). Magnetic beads were gated on their distinct scatter profile (data not shown). Early magnetic bead phagosomes were either LAMP-1^lo/−^/HLA-I^+^, LAMP-1^+^/HLA-I^lo/−^, or double negative (Figure 3B). The latter population likely represents unphagocytosed beads. Analysis of the LAMP-1^lo/−^/HLA-I^+^ population revealed phagosomes that are positive for the early endosomal markers rab5 and transferrin receptor (TfR), and strongly positive for HLA-A2 and HLA-I (Figure 3B). This population likely represents beads that have recently been phagocytosed. Consistent with this hypothesis, this population is absent following overnight incubation. Alternately, the LAMP-1^+^/HLA-I^lo/−^ population had greatly reduced levels of early endosomal markers after one hour and were essentially devoid of these markers after an overnight incubation (Figure 3B). These data demonstrate normal phagosomal maturation of inert particles.

In contrast, flow organellometry of Mtb phagosomes revealed a distinct phagosomal phenotype. After one hour of Mtb infection, the LAMP-1^lo/−^/HLA-I^+^ population seen in magnetic bead phagosomes was not detected (data not shown), possibly a reflection of the smaller number of events obtained for Mtb phagosomes. As a result, Mtb phagosomes could be accurately gated solely based on GFP and LAMP-1 (Figure 3B). Mtb phagosomes obtained one hour after infection contained similar or greater levels of early endosomal markers to those seen in the LAMP-1^+^/HLA-I^lo/−^ magnetic bead population (Figure 3C and 3D). However, overnight Mtb phagosomes remained positive for rab5, TfR, and HLA-I (Figure 3B and 3C). This phenotype is consistent with early endosomal arrest, a hallmark of the Mtb phagosomes [2].

**Figure 4 ppat-1000374-g004:**
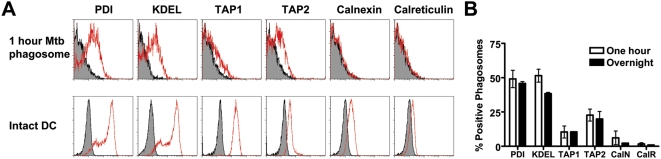
The Mtb phagosome contains HLA-I loading accessory molecules. (A) DC were pulsed with H37Rv-eGFP for 20 minutes, washed, and incubated for 40 minutes. Phagosomal fractions were prepared as in Figure 3 and stained with the indicated antibodies (top panel). Intact DC were fixed, permeabilized, and stained with the indicated antibodies (bottom panel). Data are representative of three experiments. (B) Quantitative analysis of Mtb phagosomes over time. The percent positive number represents Overton cumulative histogram subtraction of the isotype control from the indicated stain. Each bar represents the mean±SEM from three experiments per timepoint.

Having purified and phenotyped the Mtb phagosome, we asked whether HLA-I associated loading molecules were present. In addition to HLA-I (Figure 3), the Mtb phagosome contained loading accessory molecules TAP1, TAP2, and PDI, as well as proteins containing the KDEL ER retrieval sequence (Figure 4A and 4B). These proteins were found at similar levels at one hour and overnight, suggesting that these molecules are stable within the Mtb phagosome. Detection of ER proteins in the Mtb phagosome did not correlate with the total cellular levels of these proteins in DC (Figure 4A, note TAP2 versus TAP1, calnexin, and calreticulin). Furthermore, Mtb phagosomes were negative for ER-resident proteins calnexin and calreticulin. These results suggest that the presence of ER proteins was selective and not the result of gross contamination during homogenization or staining.

**Figure 5 ppat-1000374-g005:**
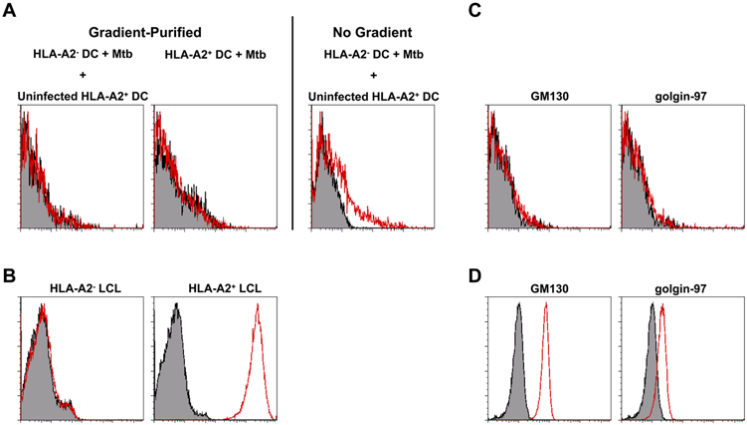
Mtb phagosomes contain minimal contamination. (A) HLA-A2^−^ or HLA-A2^+^ DC were infected with H37Rv-eGFP for 20 minutes, washed, and incubated for an additional 40 minutes. HLA-A2^−^ DC were mixed with uninfected HLA-A2^+^ DC, homogenized, and homogenate separated using 27% percoll as described or pelleted without percoll separation. Phagosomes were stained with an antibody to HLA-A2. Shaded histograms represent isotype staining. Data are representative of three experiments. (B) HLA-A2^−^ or HLA-A2^+^ LCL were fixed, permeabilized, and stained with an antibody to HLA-A2. (C,D) Mtb phagosomes (C) or intact DC (D) were analyzed for the presence of cis- and trans-golgi markers GM130 and golgin-97, respectively. Data in C are representative of three experiments each after a 40 minute or overnight chase. Data in D are representative of two experiments.

To further control for contamination, we performed a mixing experiment in which HLA-A2 could be used to discern membrane contamination. HLA-A2^−^ DC were infected with Mtb and mixed with uninfected HLA-A2^+^ DC. Following homogenization and percoll separation, phagosomes were stained with an antibody to HLA-A2. We did not detect any HLA-A2 in the Mtb phagosome after mixing (Figure 5A), suggesting that there is little non-specific association of membranes with Mtb phagosomes during processing. However, HLA-A2 contamination could be detected if phagosomes were pelleted from the postnuclear supernatant without first separating over the percoll gradient. Interestingly, while Mtb phagosomes are positive for HLA-I (Figure 3), we did not detect HLA-A2 in phagosomes from HLA-A2^+^ DC (Figure 5A). However, we were able to detect HLA-A2 staining in LAMP-1^lo/−^/HLA-I^+^ magnetic bead phagosomes (Figure 3C) and in lymphoblastoid cell lines (LCL) (Figure 5B). Because HLA-A2 is expressed at much higher levels in DC than any of the ER molecules examined, we conclude that there could only be minimal contamination of ER proteins in our phagosomal preparations.

Finally, we assessed the levels of cis- and trans-golgi markers GM130 and golgin-97, respectively. Neither protein was present in the Mtb phagosome (Figure 5C), but were detected in intact DC (Figure 5D). Together, these data suggest that HLA-I and associated loading accessory molecules are bona fide constituents of the Mtb phagosome.

### Loaded HLA-I:peptide complexes are present in the Mtb phagosome

**Figure 6:**
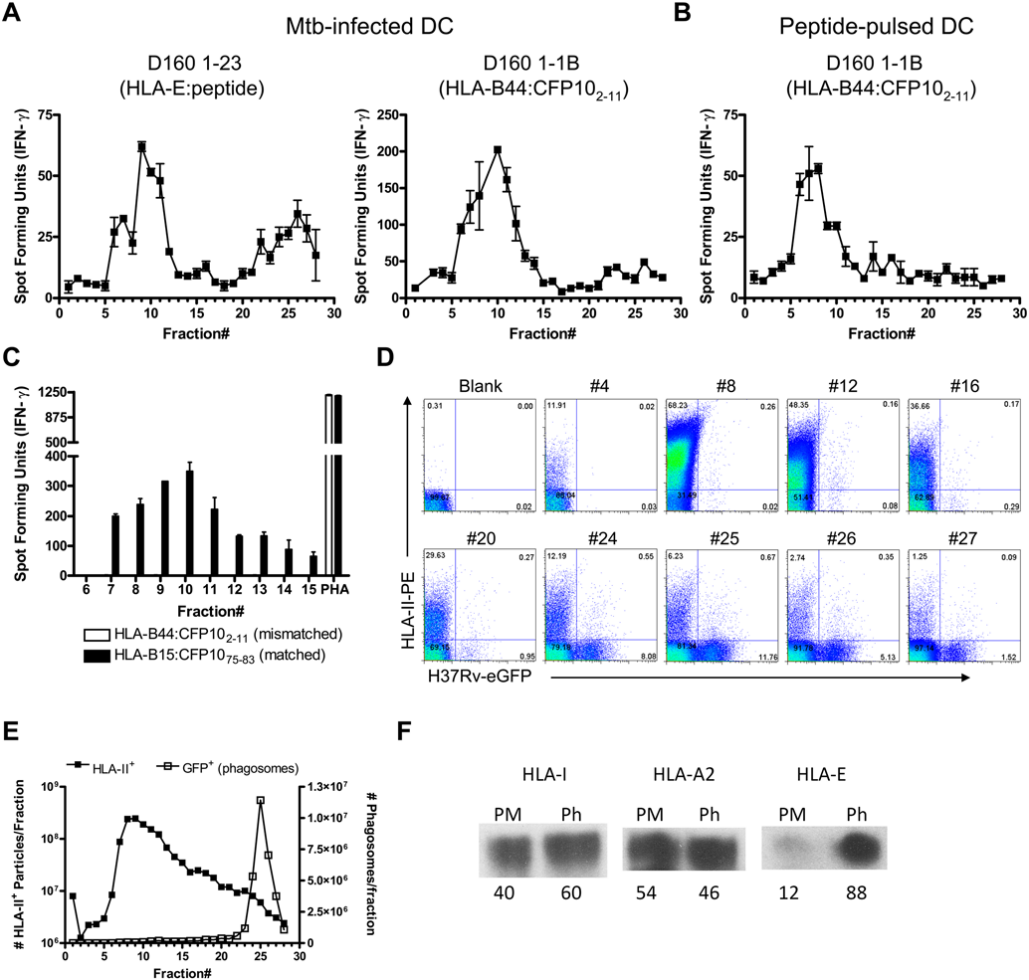
HLA-E:peptide complexes are present in phagosomal fractions. (A,B) DC were pulsed with H37Rv-eGFP or CFP10_2–11_ peptide and the homogenate was separated using 27% percoll as described. Each fraction was freeze-thawed and tested for its ability to stimulate D160 1-23 (A) or D160 1-1B (A&B) CD8^+^ T cell clones in the absence of additional APC. IFN-γ production was measured using ELISPOT. The mean±SEM of duplicate wells is presented. Data are representative of four experiments at various timepoints in A, and four experiments after a one hour peptide pulse in B. (C) Plasma membrane/ER fractions from Mtb–infected DC (HLA-B15^+^/HLA-B44^−^) were incubated with HLA-I matched or mismatched CD8^+^ T cell clones and IFN-γ production was measured using ELISPOT. The mean±SEM of duplicate wells is presented and data are representative of three experiments done similarly. (D,E) Individual fractions were analyzed by flow cytometry to assess HLA-II-PE (plasma membrane) and H37Rv-eGFP fluorescence. Selected fractions are shown (D) including the peak plasma membrane fraction (#8) and phagosomal fractions (#24–27). Prior to flow cytometry, fractions were mixed with a reference latex bead population at a known concentration. Equal numbers of latex bead events were collected for all fractions and used to quantify the number of plasma membrane and phagosome particles (E) as described in Materials and Methods. (F) HLA-I was immunoprecipitated from plasma membrane or phagosome fractions using W6/32. After dilution of the plasma membrane sample to give similar levels of HC10 staining, the presence of HLA-I alleles was assessed using antibodies to pan-HLA-I (HC10), HLA-A2 (HCA2), and HLA-E (MEM-E/02). The numbers below blots indicate the relative intensity of the bands as described in Materials and Methods.

To assess whether peptide loading occurs in the Mtb phagosome, percoll-separated fractions from Mtb–infected DC were tested for their ability to stimulate CD8^+^ T cell clones in the absence of DC by IFN-γ ELISPOT. Mtb phagosomes were purified by percoll density gradient and, as was described above, were localized to fractions 23–28 and were well-separated from plasma membrane and ER positive fractions. Percoll separated fractions were freeze-thawed to expose lumenal HLA-I, and incubated with Mtb-specific CD8^+^ T cell clones restricted by HLA-E or HLA-B44. For this experiment, we used a HLA-B44-restricted T cell clone specific for CFP10_2–11_, which we have previously shown is sensitive to BFA and lactacystin and requires TAP transport [16], similar to CFP10_3–11_. As expected, both clones responded to plasma membrane/ER fractions, revealing the presence of loaded HLA-I:peptide complexes in these cellular fractions (Figure 6A). Loaded HLA-E:peptide complexes were also detected in phagosome-containing fractions as early as one hour post-infection, suggesting that peptide loading can in fact occur within the Mtb phagosome, although we cannot formally exclude loading in another compartment and subsequent trafficking to the phagosome. Fewer HLA-B44:CFP10_2–11_ complexes were detected within phagosome fractions, suggesting that less CFP10_2–11_ loading occurs within the phagosome. These data are consistent with BFA data, showing that almost all CFP10_2–11_ processing and presentation requires ER-golgi transport [16].

To exclude the possibility that stimulation was due to contamination of ER- or plasma membrane-derived HLA-I:peptide complexes, T cells were stimulated with fractions from CFP10_2–11_ peptide-pulsed DC. No responses were detected in the high density fractions (Figure 6B). To exclude the possibility that pre-formed peptide was simply being presented by T cells, we tested fractions from Mtb–infected, HLA-I mismatched DC for their ability to stimulate Mtb-specific CD8^+^ clones. Clone D481 C10, which responds to CFP10_75–83_ in the context of HLA-B1502 [22] was stimulated by plasma membrane/ER fractions from Mtb–infected D481 DC (HLA-B15^+^/HLA-B44^−^). No HLA-B44:CFP10_2–11_ responses were detected (Figure 6C). These data suggest that HLA-I loading can occur in the Mtb phagosome and is enhanced for HLA-E.

To quantitatively assess the degree of plasma membrane contamination of the phagosome-containing fractions, plasma membrane was labeled with a PE-conjugated antibody to HLA-II prior to homogenization, and presence of plasma membrane in each fraction was assessed by flow cytometry. Phagosome-containing fractions contained some PE^+^ plasma membranes, with very little associated with phagosomes (Figure 6D, note lack of a distinct double positive population). The number of plasma membrane particles in each fraction was quantified using a reference bead population (see Materials and Methods). Consistent with data obtained in Figure 3A, the number of plasma membrane particles were strongly associated with the plasma membrane peak, and decreased in every fraction thereafter (Figure 6E). These data, then, suggest that the IFN-γ response in the phagosomal fractions cannot be attributed to gross plasma membrane contamination, a conclusion strengthened by the relative enrichment of HLA-E specific activity in the phagosomal fractions.

### HLA-E is enriched in phagosomal fractions after Mtb infection

Since there was an enhanced HLA-E specific response to the Mtb phagosome (Figure 6), we studied whether HLA-E may be preferentially present within the phagosome. After percoll separation of homogenates from Mtb–infected DC, HLA-I from plasma membrane or phagosomal fractions was immunoprecipitated with the pan-HLA-I antibody, W6/32, and HLA-I levels in each compartment were compared by western blot using three different HLA-I antibodies. Because HLA-I levels are greatly increased on the plasma membrane compared to the phagosome, the immunoprecipitate from plasma membrane was normalized to give roughly equivalent intensity bands for total HLA-I levels (HC10). While the blots for HLA-A2 are similar to that for HC10, the intensity of HLA-E is increased over seven-fold in the phagosome (Figure 6F). These data support the functional data that HLA-E complexes are more easily detected than HLA-B, and suggest that there may be preferential trafficking or retention of certain HLA-I alleles to the Mtb phagosome. Additionally, these data are not consistent with simple contamination.

### HLA-I, loading machinery, and HLA-I:peptide complexes are present in highly pure Mtb phagosomes

**Figure 7:**
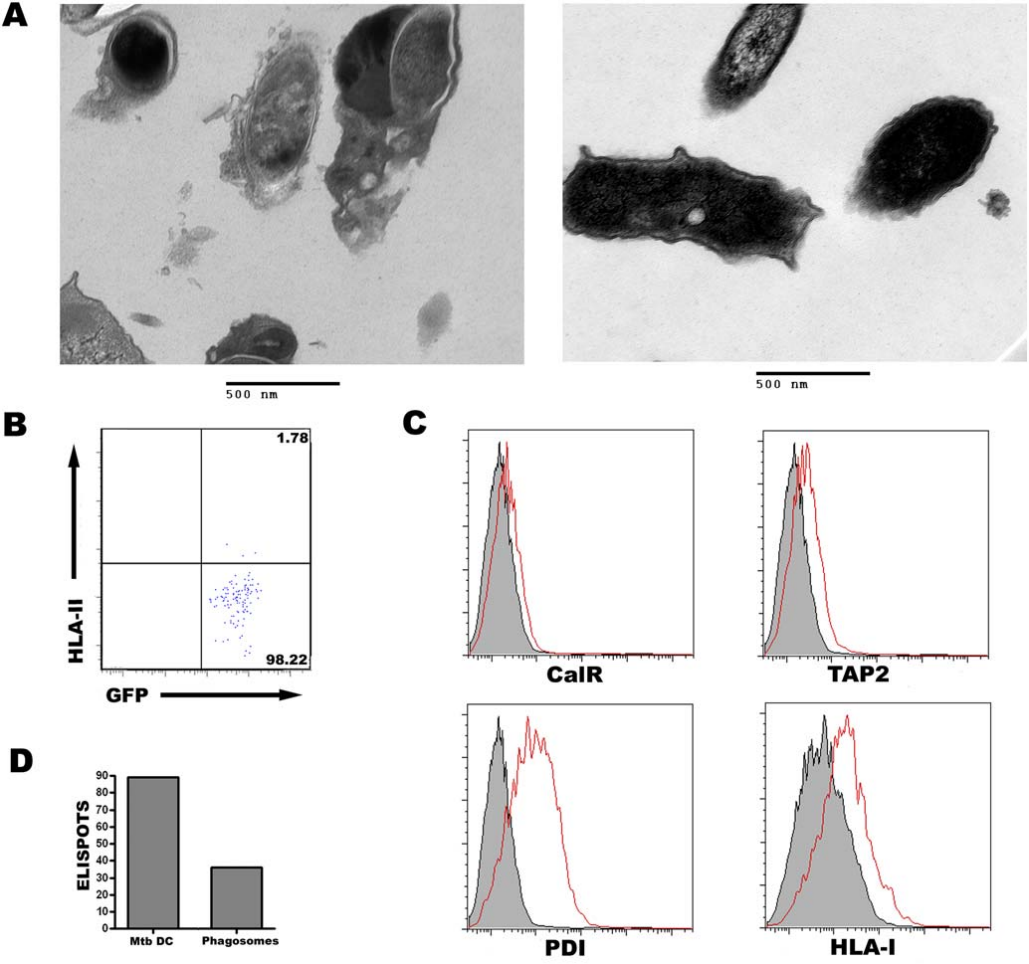
HLA-I, loading machinery, and HLA-I:peptide complexes are present in highly pure Mtb phagosomes. (A) Phagosomes were isolated by percoll gradient or magnetic purification and prepared for electron microscopy as described in Materials and Methods. (B) Magnetic bead-isolated phagosomes were analyzed by flow cytometry to assess HLA-II-PE (plasma membrane) and H37Rv-eGFP fluorescence as described in Materials and Methods and Figure 6. The events shown represent a small proportion of the population of phagosomes isolated and the experiment is representative of three experiments. (C) DC were pulsed with magnetically-labeled H37Rv-eGFP for 20 minutes, washed, and incubated for 18 hr. After magnetic separation of Mtb phagosomes, flow organellometry was performed as described previously. Data are representative of three experiments. (D) Magnetically-isolated Mtb phagosomes were freeze-thawed and tested for their ability to stimulate D160 1-23 CD8^+^ T cell clones in the absence of additional APC. IFN-γ production was measured using ELISPOT. Data are representative of two experiments.

To further control for contamination of Mtb phagosomes, a method was devised to magnetically purify Mtb phagosomes, thereby generating a highly pure phagosomal preparation. After biotinylation of Mtb cell surface proteins, the bacteria were washed and incubated with streptavidin-coated supraparamagnetic particles (approximately 50 nm diameter). DC were infected with the labeled bacteria, and phagosomes were purified by passing the postnuclear supernatant directly over a magnetic column. The resulting population is very pure as seen by electron microscopy (EM, Figure 7A), which shows the presence of Mtb surrounded by an intact phagosomal membrane and little detectible contaminating particles attached to the phagosome. The few contaminating particles present in the magnetic fraction were identified as free magnetic beads. When contamination of plasma membrane was assessed by flow organellometry, less than 2% of the purified phagosomes were associated with PE^+^ plasma membranes (Figure 7B). In comparison, 5–7% of the percoll-separated phagosomes were associated with plasma membrane (Figure 6C). Therefore, based on EM and flow organellometry, this purification protocol results in phagosomes that are at least 98% pure.

The presence of HLA-I and members of the peptide loading complex were next examined by flow organellometry. HLA-I, PDI, and TAP2 were all detected on the magnetically-purified 18-hour Mtb phagosomes, while calreticulin was excluded (Figure 7C), similar to the results presented in Figures 3–5. In total, these data establish the presence of ER proteins that aid in HLA-I presentation in the Mtb phagosome.

Finally, magnetically-purified phagosomes were used to examine the presence of loaded HLA-E:peptide complexes, as described in Figure 6. The addition of freeze-thawed, magnetically-purified Mtb phagosomes to D160 1-23 led to IFN-γ production (Figure 7D), demonstrating the presence of loaded HLA-E:peptide complexes in the Mtb phagosome. Unfortunately, the lower phagosomal yields obtained using this protocol precluded us from simultaneously testing the responses of both CD8^+^ T cell clones. Overall, these results confirm the data presented in Figures 3–6, and provide further evidence that the presence of ER proteins and loaded HLA-I:peptide complexes are not due to contaminating membranes in the percoll separated phagosomes.

## Discussion

Here, we find that presentation of both the HLA-E antigen and CFP10 require proteasomal degradation and TAP transport. Presentation of both antigens is blocked by the retrotranslocation inhibitor exoA. In contrast to presentation on HLA-B8, HLA-E presentation is only partially BFA-sensitive and is enhanced with cycloheximide treatment. We find the presence of HLA-I and ER-derived HLA-I loading accessory molecules PDI and TAP in the Mtb phagosome. Accordingly, we are able to detect loaded HLA-E:peptide complexes in the Mtb phagosome.

These data demonstrate that both classically and non-classically presented Mtb proteins require cytosolic access for presentation. Both epitopes are generated by the cytosolic proteasome and require subsequent TAP transport. The cytosolic requirement for presentation of these antigens differs from that previously shown for the presentation of the Mtb 19 kDa lipoprotein [19] and for cross-presentation of apoptotic bodies from mycobacterially-infected cells [20], demonstrating the potential for multiple presentation pathways. In this regard, the pathway by which a specific antigen is processed likely depends on the characteristics of the antigen (i.e. secreted versus non-secreted versus lipoprotein), the nature of delivery (i.e. apoptotic body versus Mtb bacilli), and specific properties of the associated HLA-I allele (see below). In the murine model, a dominant pathway has not been elucidated. We have examined the processing pathway of an additional seven HLA-I Mtb-derived epitopes and found that all are presented using the cytosolic pathway (J. Grotzke, manuscript in preparation). These findings suggest that Mtb proteins are preferentially processed in the cytosol in Mtb–infected DC.

A recent report has shown that Mtb is able to escape the phagosome after 48–96 hours of infection [31], a process which requires the Region of Difference 1 (RD1 [32]). This would give Mtb antigens access to the cytosol where they would be processed as endogenously expressed antigens. The authors hypothesized that this may increase antigen presentation. We have examined Mtb–infected DC at less than 18 hours post infection, a time when all Mtb should be present within the phagosome. Furthermore, we and others have shown that RD1 is not required for presentation of Mtb antigens on class I molecules [16],[33]. This excludes phagosomal escape as a confounding factor in our interpretations.

Our data argue that the Mtb phagosome is a compartment that aids in presentation of phagosome-derived antigens, similar to that demonstrated using latex beads [11]–[13],[34]. Presentation of both Mtb antigens examined is blocked by the retrotranslocation inhibitor exoA, suggesting that phagosomes acquire the ER-derived retrotranslocation machinery which serves to transport Mtb proteins into the cytosol. The finding that exoA blocks protein synthesis and endogenous antigen processing of vaccinia-delivered antigens is conflicting with published data using the DC-like cell line, KG-1 [11]. This discrepancy may be due to the use of a cell line versus ex vivo generated DC, as we saw only minor differences in vaccinia-eGFP expression and vaccinia-expressed antigen presentation in LCL treated with exoA (data not shown). The NAD analog PJ34 was used to uncouple the protein synthesis and retrotranslocation inhibition functions of exoA. When primary DC were co-treated with exoA and PJ34, GFP expression and endogenous antigen presentation were restored, while the inhibition of Mtb antigen presentation was unaffected. These data strongly argue that the exoA inhibition is due to retrotranslocation block, and that the retrotranslocation machinery plays a role in presentation of Mtb antigens.

The Mtb phagosome also contains members of the HLA-I peptide loading complex and loaded HLA-I:peptide complexes, suggesting that the phagosome is a site for HLA-I loading. While ICP47 inhibition demonstrates a role for TAP transport of both Mtb antigens, we cannot discern whether the blockade occurs at the ER or phagosomal membrane. The detection of TAP and loaded HLA-E in the Mtb phagosome indirectly argues that phagosomal TAP is functional. We have not addressed how ER proteins are delivered to the Mtb phagosome. Although the notion of ER-mediated phagocytosis, and particularly the relative contribution of ER membrane to the phagosomal membrane remains controversial [14],[15], multiple groups have detected members of the MHC-I peptide loading complex and functional TAP, glycosylation, and retrotranslocation machinery in latex bead phagosomes [11]–[14],[34]. Several reports have not detected the presence of ER proteins within latex bead or mycobacterial phagosomes [15],[31]. In this regard, the use of flow cytometry has enhanced our ability to find ER proteins in the Mtb phagosome, as this approach allows us to detect low protein levels and analyze thousands of events per experiment, using objective software to analyze results. Furthermore, we have included a variety of controls to address the possibility of plasma membrane or ER contamination of Mtb phagosome preparations. Our data demonstrate that the detection of HLA-I, loading accessory molecules, and HLA-I:peptide complexes are true components of the Mtb phagosome.

Although both CFP10 and the HLA-E antigen require cytosolic processing, our data demonstrate that these two antigens use distinct cytosolic pathways. After proteasomal degradation, both peptide fragments require TAP transport. CFP10_3–11_ then follows a pathway that is characteristic of presentation of endogenous proteins. In contrast, the HLA-E antigen follows a different pathway, being incompletely blocked by BFA and enhanced by cycloheximide. This argues that newly synthesized HLA-E is not required for loading, and that once loaded, HLA-E:peptide complexes do not require transport through the ER-golgi to the cell surface. In accordance with this, we detected HLA-E:peptide complexes, but fewer HLA-B44:CFP10_2–11_ complexes within the Mtb phagosome. Presentation of latex bead delivered OVA through the ER-phagosomal pathway has been shown to be only partially BFA sensitive [13], similar to the data presented here. Of note, one other group has reported BFA-independent presentation of unidentified Mtb antigen(s), although the role of the proteasome was not examined [35]. Because nascent HLA-E is not required for presentation, we believe that recycled HLA-E is loaded in the phagosome. This is similar to what has been postulated for MHC-I loading in the vacuolar pathway [9],[10]. Ackerman *et al.* reported that the majority of latex bead phagosome-associated MHC-I is endoglycosidase H resistant (post-golgi [34]). This is consistent with our hypothesis that recycled HLA-E can be loaded in the phagosome.

Our finding of divergent processing pathways during Mtb infection, and the enhanced availability of HLA-E in the phagosome suggests that HLA-E may play a specialized role in the presentation of phagosomal antigens. The ten Mtb epitopes that we have examined are presented by 8 different HLA-I alleles and we have yet to find another antigen that is as incompletely inhibited by BFA as the HLA-E antigen (J. Grotzke, manuscript in preparation [16]). Examining the requirement for new protein synthesis will allow us to further estimate the amount of phagosomal loading for each epitope and HLA-I allele. Whether HLA-E is specifically targeted to the phagosome or is delivered by enhanced recycling or is selectively retained remains to be determined. However, it is intriguing to note that HLA-E and the murine ortholog Qa-1^b^ are less stable than classical MHC-I molecules and have a higher turnover rate [36],[37]. Together, these data argue that HLA-E may uniquely participate in surveillance of phagosomal antigens.

In conclusion, we have examined in detail the presentation of two Mtb-derived antigens. We find that both antigens require cytosolic processing but differ in the overall pathway. Similar to latex bead phagosomes, the Mtb phagosome acquires components that are required for Class I presentation. These data suggest that in addition to its role in innate immunity, the phagosome aids to alert the cellular adaptive immune system to the presence of intracellular bacteria. Our data also suggest a potentially unique role for HLA-E in surveillance and presentation of phagosomal antigens. Because HLA-E exhibits low polymorphism, preferential surveillance of the phagosome by HLA-E may serve to present conserved bacterial peptides. In total, these findings demonstrate that cellular mechanisms are in place to ensure that antigens from pathogens that reside in the phagosome are not excluded from the Class I pathway.

## Materials and Methods

### Reagents and antibodies

Inhibitors of antigen processing were obtained from EMD Biosciences (epoxomicin, bafilomycin, and PJ34), Sigma (cycloheximide and *Pseudomonas aeruginosa* exotoxin A), and Invitrogen (brefeldin A). The following antibodies were used for flow cytometry: TAP1, TAP2, EEA-1, rab5, GM130, HLA-A2, LAMP-1-PE (BD Biosciences), PDI, KDEL, calreticulin (Stressgen), HLA-I-FITC or unconjugated (B9.12.1, Coulter), TfR (Biosource), calnexin (Affinity Bioreagents), golgin-97 and goat anti-mouse IgG Alexa Fluor-647 (Invitrogen). Soluble CFP10 protein was provided by Corixa. CFP10 peptides were synthesized by Genemed Synthesis Inc. Magnetic particles (2.8 µm tosylactivated beads, Dynal) were coupled to BSA according to the manufacturer's protocol before addition to DC.

### Bacteria, virus, and cells

*Mycobacterium tuberculosis* H37Rv-eGFP was grown in Middlebrook 7H9 broth supplemented with Middlebrook ADC (Fisher), 0.05% Tween-80, 0.5% glycerol, and kanamycin (50 µg/ml). Before infection, bacteria were sonicated, passaged 15 times through a tuberculin syringe, and sonicated again to obtain a single cell suspension.

Adenoviral vectors [25] were provided by Dr. David Johnson (OHSU). Vaccinia virus expressing HIV p24 was provided by Therion Biologics Corp. and vaccinia virus expressing eGFP was provided by Corixa.

This study was conducted according to the principles expressed in the Declaration of Helsinki. The study was approved by the Institutional Review Board of Oregon Health & Sciences University and the Portland VA hospitals (IRB00000186). All patients provided written informed consent for the collection of samples and subsequent analysis. PMBC were obtained from normal human donors via leukapheresis according to IRB approved protocols and processed as previously described [23]. DC were generated by culturing adherent PBMC for 5 days in the presence of GM-CSF (10 ng/ml, Amgen) and IL-4 (10 ng/ml, R&D systems) in RPMI (Gibco) supplemented with 10% pooled human serum (HS), L-glutamine (4 mM, Gibco), and gentamicin (50 µg/ml, Invitrogen).

### T cell clones

Clones D160 1-1B, D160 1-23, D480 F6, and D481 C10 have been previously described [22],[23],[38]. D454 E12 is a CFP10-specific CD4^+^ clone (unpublished). Clone 16A7 is a HLA-B44 restricted CD8^+^ T cell clone that responds to amino acids 306–316 (AEQASQEVKNW) of HIV p24 [39]. T cell clones were expanded as previously described [16], except that some of the expansions were done in Stemline T cell expansion media (Sigma) supplemented with 1% FBS (Hyclone) and 4 mM L-glutamine.

### ELISPOT Assay

IFN-γ ELISPOT was performed as previously described [23].

### Inhibition of antigen presentation

Day 5 DC were plated in 24 well ultra low adherence (ULA) plates (Costar) at 5×10^5^/well in RPMI/10% HS supplemented with GM-CSF and IL-4. DC were pretreated with inhibitors (1 µM epoxomicin, 0.1 µg/ml BFA, 0.2 µM bafilomycin, 10 µg/ml cycloheximide, 10 µg/ml exoA, or 10 µg/ml exoA with 200 fold molar excess PJ34) for one hour before infection with H37Rv-eGFP (MOI = 20), vaccinia virus (MOI = 2), or addition of antigen (0.5–1 µg/ml CFP10, 1 µg/ml CFP10_3–11_). In experiments using exoA/PJ34 or BSA/PJ34, these compounds were co-incubated at room temperature for 30 minutes before addition to DC.

DC were harvested after 15–16 hours of infection, pelleted and fixed with 0.5% paraformaldehyde for 15 minutes. The reaction was stopped with an equal volume of 0.4 M Lysine or RPMI/10% HS and DC were washed extensively with RPMI/10% HS. Fixed DC were then added to an IFN-γ ELISPOT plate at varying quantities so that antigen was the limiting factor (25,000 Mtb–infected DC/well for CD8^+^ clones, 1,000 Mtb–infected DC/well for CD4^+^ clones, and 2,000 Ag-pulsed DC/well for both CD4^+^ and CD8^+^ clones). This MOI and number of DC per well was used to obtain strong T cell responses which are still able to be inhibited with blockers of antigen presentation, as previously reported [16],[40]. T cells clones were added in excess at 10,000/well and plates were incubated at 37°C for 18 hours before development.

### ICP47-mediated TAP inhibition

DC were tranduced with adenovirus using Lipofectamine 2000 (Invitrogen) as previously described [16]. After 6–26 hours of incubation with adenovirus, media was removed and replaced with RPMI/10% HS containing H37Rv-eGFP (MOI = 15–20 for CD8^+^ T cell clones, 0.2–20 for CD4^+^ clones) or antigen (0.25 µg/ml CFP10_3–11_, 0.005 µg/ml CFP10) and incubated overnight at 37°C. After 15–16 hours, media was removed and T clones were added (1.5×10^5^/well) in the presence of BFA (10 µg/ml). After a six hour stimulation, T cell clones were harvested, fixed with 1% paraformaldehyde, and stained with antibodies to CD3 (UCHT1, BD Biosciences) and IFN-γ (Coulter) in the presence of 0.2% saponin (Sigma). Cells were analyzed using a FACSCalibur or LSR II (Becton Dickinson).

### Flow organellometry

DC were added to a 6 well ULA plate (2.5×10^6^/well) in RPMI/10% HS supplemented with GM-CSF (10 ng/ml) and IL-4 (10 ng/ml) and allowed to adhere. H37Rv-eGFP (MOI = 5–7.5) or magnetic beads (1–10/cell) were added to wells and centrifuged onto DC at 22°C for 5 min. DC were then incubated for an additional 15 min at 37°C for a total pulse time of 20 min. DC were placed on ice and washed with cold RPMI and incubated for an additional 40 min or overnight at 37°C. DC were washed on ice with cold RPMI and harvested. DC were resuspended in ice cold homogenization buffer (0.25 M sucrose, 10 mM Hepes, pH = 7.4) containing protease inhibitors (Complete Mini protease inhibitor cocktail tablet, Roche) and homogenized by passaging through a 23 gauge needle. At this point, magnetic beads were purified using a particle concentrator (Dynal). For Mtb phagosomes, intact cells and nuclei were removed by two spins at 200×g, 4°C. Postnuclear supernatant was layered onto 27% percoll (Amersham) and centrifuged for one hour at 4°C, 36,000×g in a 70.1Ti rotor (Beckman).

The phagosome-containing fractions (final 2 ml of gradient) were pelleted, fixed with 1% paraformaldehyde on ice for 15 minutes, washed, and stained with primary antibodies (5 µg/ml) for 40 min on ice in the presence of 0.2% saponin, 2% HS, 2% goat serum, and 0.5% FBS. After washing, goat anti-mouse-IgG-Alexa Fluor-647 was added (1∶1000) and incubated for 40 min. Phagosomes were then washed twice and stained with anti-LAMP-1-PE and anti-HLA-I-FITC for 40 min. Phagosomes were washed and analyzed using a FACSCalibur or LSR II.

The percentage of positive phagosomes was determined using the Overton histogram subtraction method in Flowjo software (Treestar), which compares the isotype- and experimentally-stained populations. The values obtained from this analysis are shown in Figures 3 and 4. We also analyzed each experiment by setting the isotype gate so that 5% of the events are positive, and then applied this gate to stained samples. After subtracting the isotype value, these numbers gave almost identical results as that seen using Overton analysis (data not shown).

### Subcellular fractionation

DC were plated in 6 well ULA plates as above. H37Rv-eGFP (MOI = 7.5) was added to wells and centrifuged onto DC for 5 min at 22°C. DC were incubated for an additional hour or overnight, without washing. After infection, DC were placed on ice, washed with cold RPMI, and harvested. Before homogenization, DC were labeled with a PE-conjugated antibody to HLA-II (Coulter) to track plasma membrane. Excess antibody was removed by centrifugation, cells were resuspended in homogenization buffer, homogenized and separated on a percoll gradient as above. The percoll gradient was manually fractionated into 28 fractions (332 µl each).

Lysosomes were detected using an enzymatic assay to detect β-hexosaminidase activity as previously described [30]. ER containing fractions were identified using western blot for PDI and TAP1 (Stressgen). For plasma membrane and phagosome detection, fractions were fixed with paraformaldehyde. Plasma membrane was detected using a fluorometer with an excitation filter of 530±12.5 nm and emission filter of 590±17.5 nm. Phagosomes were detected using a flow-based assay, where fixed fractions were mixed with a reference population of 2 µm latex beads (Polysciences) at a known concentration and analyzed using a FACSCalibur or LSR II. Phagosomes were detected based on GFP fluorescence and quantified using the ratio of GFP^+^ events to latex beads. Plasma membrane was also quantified using this assay and gave similar results to fluorometer data. Quantification of organelles was done using the following equation: ratio of PE^+^ or GFP^+^ to latex beads * #beads/ml * dilution factor * fraction volume

For detection of loaded HLA-I:peptide complexes, fractions were frozen overnight at −80°C and thawed. 20–45 µl fractions were added to duplicate wells of an ELISPOT plate containing 20,000 T cell clones and incubated for 18–48 hours at 37°C before development.

### Magnetic purification of Mtb phagosomes

Magnetic labeling of H37Rv-eGFP was carried out as follows. After two washes in PBS, H37Rv-eGFP was incubated with sulfo-NHS-biotin (1 mg/ml, Pierce) in PBS for 30 minutes at room temperature. The reaction was stopped with 0.1 M glycine and the bacteria washed twice in PBS/0.05% Tween-80. After 5–10 passages through a 27 gauge needle, the biotinylated H37Rv-eGFP was incubated with streptavidin-coated microbeads (Miltenyi) for 20 minutes at room temperature. The bacteria were pelleted and again passaged 5–10 times through a 27 gauge needle to create a single cell suspension. DC were infected, harvested, and homogenized as above. Phagosomes were purified by passing the postnuclear supernatant over a Miltenyi MS column on ice. After 3 washes with ice cold PBS, the bound phagosomes were eluted in 0.6 ml PBS as per the manufacturer's instruction. The purified phagosomes were used for flow organellometry or in functional assays as described above.

For EM analysis of percoll and magnetically-isolated phagosomes, pelleted samples were fixed in 100 mM sodium cacodylate (pH 7.2), 2.5% glutaraldehyde, 1.6% paraformaldehyde, 0.064% picric acid, 0.1% ruthenium red, gently washed, and postfixed for 1 hour in 1% osmium tetroxide plus 08% potassium ferricyanide in 100 mM sodium cacodylate, pH 7.2. After thorough rinsing in water, samples were prestained in 4% uranyl acetate for 1 hour, thoroughly rinsed, dehydrated, infiltrated overnight in 1∶1 acetone∶Epon 812, infiltrated 1 hour with 100% Epon 812 resin, and embedded in the resin. After polymerization, 60- to 80-nm thin sections were cut on a Reichert ultramicrotome, stained 5 min in lead citrate, rinsed, poststained 30 min in uranyl acetate, rinsed, and dried. EM was performed at 60 kV on a Philips Morgagne TEM, equipped with a CCD, and images were collected at original magnifications of 1,000–20,000×.

### Western blot analysis

After fractionation of percoll-separated postnuclear supernatant from Mtb–infected DC, fractions containing plasma membrane (fractions 7–9) or phagosomes (fractions 20–30) were resuspended in RIPA buffer for lysis. The supernatant was pre-cleared with isotype antibody (IgG2a, Biolegend) and Protein A/G beads (Calbiochem) for 1 hour each. Samples were spun and supernatant incubated with pan-HLA-I antibody (W6/32, AbD Serotec) and Protein A/G beads for 1 hour. These immunoprecipiated samples were then electrophoresed and the proteins blotted onto a polyvinylidene difluoride membrane (Immobilon-P; Millipore) and probed with the following antibodies: pan-HLA-I (HC10, kind gift from Dave Johnson, OHSU), HLA-A2 (HCA2, kind gift from Peter Creswell, Yale University and Emmanuel Wiertz, Leiden University), and HLA-E (MEM-E/02, Abcam). They were then detected using an enhanced chemiluminescence (ECL) kit (Perkin-Elmer Life Sciences). Gels were scanned into TIFF images and analyzed via Image J software (available online from National Institutes of Health).

### Statistical analysis

Statistical significance of inhibition was determined using Student's two-tailed t test compared to control-treated cells, unless otherwise indicated.
